# Assessment of Confidence, Guideline Adherence, and Resuscitation Challenges Following Advanced Cardiovascular Life Support Certification Among Postgraduate Residents: A Cross-Sectional Study

**DOI:** 10.7759/cureus.111783

**Published:** 2026-06-30

**Authors:** Chinnam Vishnupriya, Anagani Hrushikesh, Annet Maria Thomas, Devika Saju, Sreekrishnan Trikkur, Dhanasekaran BS, Gireesh Kumar

**Affiliations:** 1 Emergency Medicine, Amrita Institute of Medical Sciences, Kochi, IND; 2 Biostatistics, Amrita Institute of Medical Sciences, Kochi, IND

**Keywords:** acls training, cardiac arrest, cpr feedback devices, ecg interpretation, etco₂ monitoring, postgraduate residents, resuscitation competence, self-efficacy, simulation-based medical education

## Abstract

Background: Cardiac arrest remains a leading cause of mortality worldwide. Advanced Cardiovascular Life Support (ACLS) training is designed to equip postgraduate (PG) residents with the competencies required for effective resuscitation. However, gaps in skill retention, electrocardiogram (ECG) interpretation, and utilization of physiological monitoring tools such as end-tidal carbon dioxide (EtCO₂) monitoring and real-time cardiopulmonary resuscitation (CPR) feedback devices continue to impede resuscitation quality in clinical practice.

Aim: The aim of the study is to assess post-training confidence, perceived clinical competence, and guideline adherence among PG residents and to identify challenges encountered during live in-hospital cardiac arrest resuscitations.

Methods: A cross-sectional, feedback-based study was conducted at a tertiary care, American Heart Association (AHA)-accredited teaching hospital in South India over six months. A total of 187 PG residents from 32 departments who had completed ACLS training and participated in at least one in-hospital cardiac arrest resuscitation were enrolled. A validated, structured 24-item questionnaire (Cronbach’s α = 0.814) was administered electronically. This study was reported in accordance with the STROBE (Strengthening the Reporting of Observational Studies in Epidemiology) checklist for cross-sectional studies. Statistical analysis included descriptive statistics, Mann-Whitney U test, Spearman correlation, and multiple linear regression.

Results: Mean confidence and perceived competence scores were 9.75 ± 1.33 (95% confidence interval (CI): 9.55-9.94) and 22.83 ± 3.29 (95% CI: 22.35-23.31), respectively (all values self-reported). Significant gaps in monitoring tool utilization were identified, including non-use of real-time CPR feedback devices, absence of EtCO₂ monitoring, and difficulties with ECG rhythm interpretation during live resuscitations. Specifically, 43.5% (n = 80) of participants did not use real-time CPR feedback devices, and 48.9% (n = 89) did not utilize EtCO₂ monitoring during resuscitation. The most prevalent resuscitation challenge was ECG rhythm interpretation, reported by 34.2% (n = 64; 95% CI: 27.3%-41.9%) of participants. ACLS training quality was rated as excellent by 73% (n = 137; 95% CI: 65.8%-79.3%) of participants. No significant intergroup differences were found between clinicians and non-clinicians (p > 0.05). Confidence was the only significant predictor of perceived clinical competence (β = 0.724, p < 0.001; R² = 0.527).

Conclusion: Participants reported high levels of confidence and perceived clinical competence following ACLS training, suggesting the program provides a solid foundation for emergency cardiovascular care. Nonetheless, persistent deficiencies in ECG interpretation, feedback device utilization, and EtCO₂ monitoring adherence warrant targeted curricular interventions, including structured ECG modules, biannual simulation-based refresher training, and mandatory integration of physiological monitoring tools. Future multicenter studies with objective performance metrics are recommended.

## Introduction

Cardiac arrest is a life-threatening emergency characterized by the sudden cessation of effective cardiac activity, resulting in loss of consciousness, absence of a palpable pulse, and apnea [1]. Despite significant advances in resuscitation science over the past two decades, cardiac arrest remains a leading cause of mortality globally. In-hospital cardiac arrest (IHCA) carries a survival-to-discharge rate of approximately 20%-25% [2], while out-of-hospital cardiac arrest (OHCA) outcomes are markedly more sobering, with survival rates consistently reported to be below 10% in most series [3]. Long-term cerebral hypoxia frequently results in poor neurological outcomes even among survivors, further underscoring the public health importance of high-quality, timely resuscitation.

Effective management of cardiac arrest is contingent upon the prompt, coordinated execution of the American Heart Association (AHA) Chain of Survival, encompassing early recognition, activation of the emergency response system, high-quality cardiopulmonary resuscitation (CPR), rapid defibrillation, advanced life support, and structured post-cardiac arrest care [4]. Any deficiency or delay in these sequential steps substantially reduces the probability of survival and favorable neurological recovery. Postgraduate (PG) medical residents frequently serve as first responders or resuscitation team leaders during IHCA events; their competency in Advanced Cardiovascular Life Support (ACLS) protocols is therefore directly linked to patient outcomes [5].

In response to the need for standardized resuscitation training, the AHA developed the ACLS curriculum. ACLS training encompasses airway management, cardiac rhythm recognition, pharmacological intervention, defibrillation, and coordinated team-based care. Institutions that achieve AHA accreditation are required to deliver structured resuscitation training as a mandatory component of PG medical curricula [5]. However, several well-documented challenges continue to limit the real-world effectiveness of ACLS training, including skill decay, gaps in electrocardiogram (ECG) interpretation proficiency, and inconsistent utilization of advanced physiological monitoring tools such as end-tidal carbon dioxide (EtCO₂) monitors and real-time CPR feedback devices [6,7].

Skill retention following ACLS certification is a recognized concern. Systematic reviews have consistently demonstrated that critical cognitive and psychomotor competencies, including chest compression quality, advanced airway management, and arrhythmia recognition, decline significantly in the absence of structured, repetitive practice. More than 50% of certified providers have been reported to fail competency benchmarks within six months of certification, particularly in high-stress, low-frequency scenarios such as cardiac arrest [6]. Smith et al. similarly demonstrated that both ACLS and Basic Life Support (BLS) skill retention among hospital staff declined significantly within months of initial certification, with pass rates for critical skills dropping below 50% within six months of training [8]. ECG interpretation represents a further area of vulnerability: despite its centrality to ACLS algorithms, the standard curriculum does not include a dedicated ECG module, leaving many residents underprepared for rhythm-based clinical decision-making under pressure [9].

The AHA recommends the integration of advanced physiological monitoring tools, specifically EtCO₂ monitoring and real-time audiovisual CPR feedback devices, into in-hospital resuscitation practice. EtCO₂ levels during CPR provide a non-invasive, objective surrogate of cardiac output and pulmonary blood flow and constitute the earliest detectable physiological marker of return of spontaneous circulation (ROSC) [10,11]. Real-time feedback devices facilitate continuous, dynamic correction of compression depth, rate, and recoil, promoting consistent adherence to AHA guideline targets [12]. Despite robust evidence supporting their clinical benefit, utilization of these tools in clinical practice remains inconsistent [12]. The 2015 AHA guidelines explicitly emphasize CPR quality metrics, including compression rate, depth, recoil, and minimization of interruptions as modifiable determinants of survival, and mandate the use of real-time feedback systems to ensure consistent adherence to these standards during both training and clinical resuscitation [13].

This article was previously presented as a poster at the Department of Emergency Medicine Annual Research Day, Amrita Institute of Medical Sciences, Kochi, in January 2026. Given the pivotal role of PG residents in resuscitation teams and the well-documented limitations of the current ACLS training program, this study aimed to evaluate post-training confidence, perceived clinical competence, and guideline adherence among PG medical residents at an AHA-accredited tertiary care center. A secondary objective was to systematically identify the challenges encountered during live IHCA resuscitations, with particular attention to ECG interpretation difficulties and the utilization of EtCO₂ monitoring and CPR feedback devices. The findings are intended to inform evidence-based recommendations for improving ACLS training curricula and resuscitation practice standards.

## Materials and methods

Study design

A cross-sectional, feedback-based study was conducted to evaluate the impact of ACLS training on PG medical residents. This study was reported in accordance with the STROBE (Strengthening the Reporting of Observational Studies in Epidemiology) checklist for cross-sectional studies. Primary outcomes included self-reported confidence in resuscitation procedures, perceived clinical competence, adherence to AHA resuscitation protocols, and the nature of challenges encountered during actual cardiac arrest events. Data were collected at a single time point, approximately six months after the completion of formal ACLS training, to evaluate self-reported confidence and perceived applicability of acquired skills under clinical conditions. ACLS training at this institution was conducted in multiple batches throughout the academic period, with each batch facilitated by certified AHA faculty using identical course materials and standardized case scenarios based on the 2020 AHA guidelines, thereby ensuring uniformity in training content and assessment standards across all batches. Participants' ACLS certification completion dates were self-reported via item Q4 of the study questionnaire and subsequently cross-verified against the institution's official training records to confirm that a minimum of six months had elapsed between each participant's certification date and the date of questionnaire administration. Only residents whose records confirmed eligibility within this post-certification window were included in the final analysis.

Study setting

The study was conducted at Amrita Institute of Medical Sciences, a tertiary care teaching hospital in Kochi, Kerala, South India. The institution operates a high-capacity emergency department and fully functional intensive care units and holds accreditation as an AHA ACLS training center. Structured resuscitation training is a mandatory component of the PG medical curriculum at this institution. The study was conducted from July 17, 2024, to December 29, 2024. The study period spanned six months following the conclusion of the ACLS training batches conducted during the academic period, thereby allowing adequate time for participants to encounter resuscitation scenarios in their respective clinical rotations.

Study population and eligibility criteria

The study population comprised 187 PG medical residents who had completed AHA-accredited ACLS training in accordance with the 2020 AHA guidelines. All participants completed the same version of the AHA ACLS Provider Course based on the 2020 AHA Guidelines for CPR and Emergency Cardiovascular Care, delivered using standardized course materials and identical case scenarios across all training batches, with sessions facilitated by the same pool of certified AHA ACLS instructors, thereby ensuring uniformity of training content and delivery across all participants.

Inclusion criteria required that participants (i) had completed ACLS training at the study institution, (ii) were enrolled in a PG medical program at the time of the study, and (iii) had been directly involved in at least one IHCA resuscitation event within six months of completing ACLS training. Residents who had not participated in any resuscitation event during the study period, as well as those who did not complete the study questionnaire, were excluded from the final analysis. It is acknowledged that this inclusion criterion may have introduced selection bias, as residents who had not encountered a resuscitation event, potentially those in less acute or procedurally inactive rotations, were systematically excluded, which may have resulted in a study sample with comparatively higher clinical exposure than the broader PG resident population. This limitation is discussed further in Limitations.

A total of 217 PG residents submitted responses to the electronic questionnaire during the data collection period (August 2024-December 2024). Of these, 30 were excluded from the final analysis as they did not meet the inclusion criteria, primarily due to non-participation in any IHCA resuscitation event during the study period or incomplete questionnaire submission, yielding a final analytical sample of 187 participants, representing an inclusion rate of 86.2% of all respondents. As data collection was administered anonymously through a secure, password-protected online platform, formal tracking of individual non-responders was not feasible, and a precise response rate relative to the total eligible population could not be calculated; this is acknowledged as a limitation of the anonymous data collection approach.

Written informed consent was obtained from all participants prior to enrollment. The mean interval between ACLS certification completion and questionnaire administration was approximately six months (range: six to 12 months), as data collection was conducted at the conclusion of the six-month study period following the ACLS training batches conducted during the academic year. This interval was selected to allow adequate clinical exposure while capturing skill retention within a timeframe consistent with published reports of ACLS skill decay [6,8].

Sample size

The sample size was estimated on the basis of pilot data, yielding a monitoring tool non-utilization proportion of approximately 45%. Using a 10% margin of error and a 95% confidence interval (CI), the minimum required sample was calculated at 95 participants using Cochran’s formula (n = Z² × p(1 − p)/e², where Z = 1.96, p = 0.45, and e = 0.10) [14]. The final study sample of 187 eligible residents who met all inclusion criteria substantially exceeded this minimum threshold, thereby ensuring adequate statistical power for all planned analyses.

Data collection instrument

Content validity of the questionnaire was established through a structured expert review process. A panel of five subject matter experts was convened, comprising three senior faculty members from the Department of Emergency Medicine, one specialist from Critical Care Medicine, and one faculty member from Medical Education, each with a minimum of five years of active involvement in resuscitation training and curriculum development. Experts independently reviewed each item for relevance, clarity, and representativeness of the construct being measured and provided written feedback. Items were revised, reworded, or removed based on consensus agreement across at least four of the five panel members, following two iterative rounds of review.

A pilot study was subsequently conducted with 15 PG residents drawn from clinical and non-clinical departments who were not included in the main study sample. Pilot testing assessed item clarity, comprehension, and response feasibility. Based on pilot feedback, three items were reworded to improve clarity in the Barriers and Challenges domain, and one response option was added to item Q14 (calculation of chest compression fraction) to capture non-calculating respondents. Nevertheless, the multi-stage development process incorporating expert panel review, iterative item refinement, and pilot testing provides a reasonable basis for content validity, and the acceptable Cronbach's alpha (0.814) supports the internal consistency of the instrument.

Scoring Methodology

The Confidence in ACLS Application domain (Q5-Q9) was scored on a five-point Likert scale (1 = lowest, 5 = highest), yielding a maximum domain score of 12 after recoding. The Perceived Clinical Competence domain (Q10-Q16) yielded a maximum composite score of 28. The Monitoring and Adherence domain yielded a maximum score of 8. The Barriers score reflected a count of challenges selected from 11 predefined domains (range 0-14). Internal consistency was assessed using Cronbach's alpha, which yielded a value of 0.814, indicating acceptable reliability [15]. The two physiological monitoring tools assessed in this study were (1) real-time CPR feedback devices, which provide audiovisual feedback on compression depth, rate, and recoil during resuscitation, and (2) EtCO₂ monitoring systems, which serve as non-invasive surrogates of cardiac output and early indicators of ROSC. The complete 24-item questionnaire is provided in Appendix A. The final questionnaire was administered electronically via Google Forms (Google, Mountain View, CA, USA), and all responses were anonymized and stored in a password-protected institutional database.

Statistical analysis

Continuous variables are reported as mean ± standard deviation (SD) with 95% CI. Categorical variables are expressed as frequency and percentage with 95% CI. Prior to inferential analysis, normality of distribution was assessed using the Shapiro-Wilk test. Given evidence of non-normal distribution across all key variables (p < 0.001), the Mann-Whitney U test was applied for between-group comparisons of clinicians versus non-clinicians. Spearman’s rank correlation coefficient (ρ) was used to assess the relationship between confidence and perceived clinical competence. Multiple linear regression analysis was performed to identify independent predictors of perceived clinical competence, with confidence score, barriers score, group classification, EtCO₂ monitoring use, and ECG interpretation difficulty as predictor variables. Prior to regression modeling, standard assumptions were examined: linearity was assessed via partial regression plots, independence of residuals via the Durbin-Watson statistic, homoscedasticity via visual inspection of standardized residual plots, normality of residuals via the Kolmogorov-Smirnov test, and multicollinearity via variance inflation factor (VIF) values, with a threshold of VIF < 5 considered, and influential outliers were assessed using Cook's distance, with a threshold of 4/n considered acceptable. Although perceived clinical competence is a composite Likert-based score, its treatment as a continuous variable in linear regression is consistent with established practice in health education research [16]. A two-tailed p-value of <0.05 was considered statistically significant. All analyses were performed using IBM SPSS Statistics Version 20.0 (IBM Corp., Armonk, NY, USA).

Ethical considerations

The study was conducted in accordance with the Declaration of Helsinki. Ethical approval was obtained from the Amrita Institute of Medical Sciences Institutional Ethics Committee (Ethics Approval No. ECASM-AIMS-2024-581). Participation was voluntary, and written informed consent was obtained from all participants prior to enrollment. Anonymity and confidentiality of all responses were maintained throughout the study. A copy of the informed consent form, along with one completed and signed instance obtained from a study participant, is provided in Appendix B for reference and records.

## Results

All outcome measures in this study are based on self-reported questionnaire data and should be interpreted accordingly.

Participant characteristics

A total of 187 PG medical residents completed the study questionnaire, representing 32 clinical and non-clinical departments. Of the 217 residents who submitted responses, 30 were excluded from the final analysis as they did not meet the inclusion criteria, primarily due to non-participation in any IHCA resuscitation event during the study period or incomplete questionnaire submission, yielding an inclusion rate of 86.2% of all respondents. Of the final 187 participants, 133 (71.1%) were from clinical specialties and 54 (28.9%) were from non-clinical or para-clinical departments. The largest departmental contributions were from Pathology (n = 19; 10.2%), Radiodiagnosis (n = 16; 8.6%), Anesthesia (n = 15; 8.0%), Emergency Medicine (n = 11; 5.9%), Community Medicine (n = 11; 5.9%), and General Surgery (n = 11; 5.9%). The complete distribution of participants across all departments is presented in Table 1.

**Table 1:** Distribution of postgraduate resident participants across departments (N = 187). Departments are listed alphabetically; clinical and non-clinical subtotals are shown. OBG: Obstetrics and Gynecology; ENT: Ear, Nose, and Throat

Department/specialty	N	%
Anesthesia	15	8.0
Anatomy	1	0.5
Biochemistry	4	2.1
Cardiac Anesthesia	1	0.5
Cardiology	2	1.1
Clinical Hematology	1	0.5
Community Medicine	11	5.9
Dermatology	4	2.1
Emergency Medicine	11	5.9
ENT	7	3.7
Forensic Medicine & Toxicology	6	3.2
General Medicine	6	3.2
General Surgery	11	5.9
Microbiology	6	3.2
Nephrology	1	0.5
Neurosurgery	3	1.6
Nuclear Medicine	5	2.7
OBG	9	4.8
Ophthalmology	9	4.8
Orthopedics	3	1.6
Pediatric Neurology	2	1.1
Palliative Medicine	3	1.6
Pathology	19	10.2
Pediatrics	4	2.1
Pharmacology	2	1.1
Physical Medicine & Rehabilitation	5	2.7
Physiology	5	2.7
Psychiatry	5	2.7
Radiation Oncology	4	2.1
Radiodiagnosis	16	8.6
Respiratory Medicine	4	2.1
Rheumatology	2	1.1
Clinical Departments subtotal	133	71.1
Non-clinical Departments subtotal	54	28.9
Total	187	100.0

With respect to the year of PG residency, the majority of participants were in their third year or above (n = 97; 48.0%), followed by first-year residents (n = 77; 38.1%), second-year residents (n = 21; 10.4%), and a small number whose year of training could not be unambiguously classified from the free-text entry (n = 7; 3.5%). Age and gender were not collected as discrete variables in the study instrument, as the questionnaire was designed to maintain full participant anonymity in accordance with the ethical framework of the study; this omission is acknowledged as a limitation. All participants completed the same AHA ACLS Provider Course based on the 2020 AHA Guidelines for CPR and Emergency Cardiovascular Care, delivered using standardized course materials and identical case scenarios across all training batches, with sessions facilitated by the same pool of certified AHA ACLS instructors. A comprehensive summary of baseline participant demographic and training characteristics is presented in Table 2.

**Table 2:** Baseline demographic and training characteristics of postgraduate resident participants by year of residency and department classification (N = 187).

Characteristic	n	%
Year of postgraduate residency		
First year	77	38.1
Second year	21	10.4
Third year or above	97	48.0
Not specified	7	3.5
Subtotal	202	100.0
Department classification		
Clinical specialties	133	71.1
Non-clinical/para-clinical	54	28.9
Total	187	100.0

Descriptive statistics of key study variables

**Figure 1:**
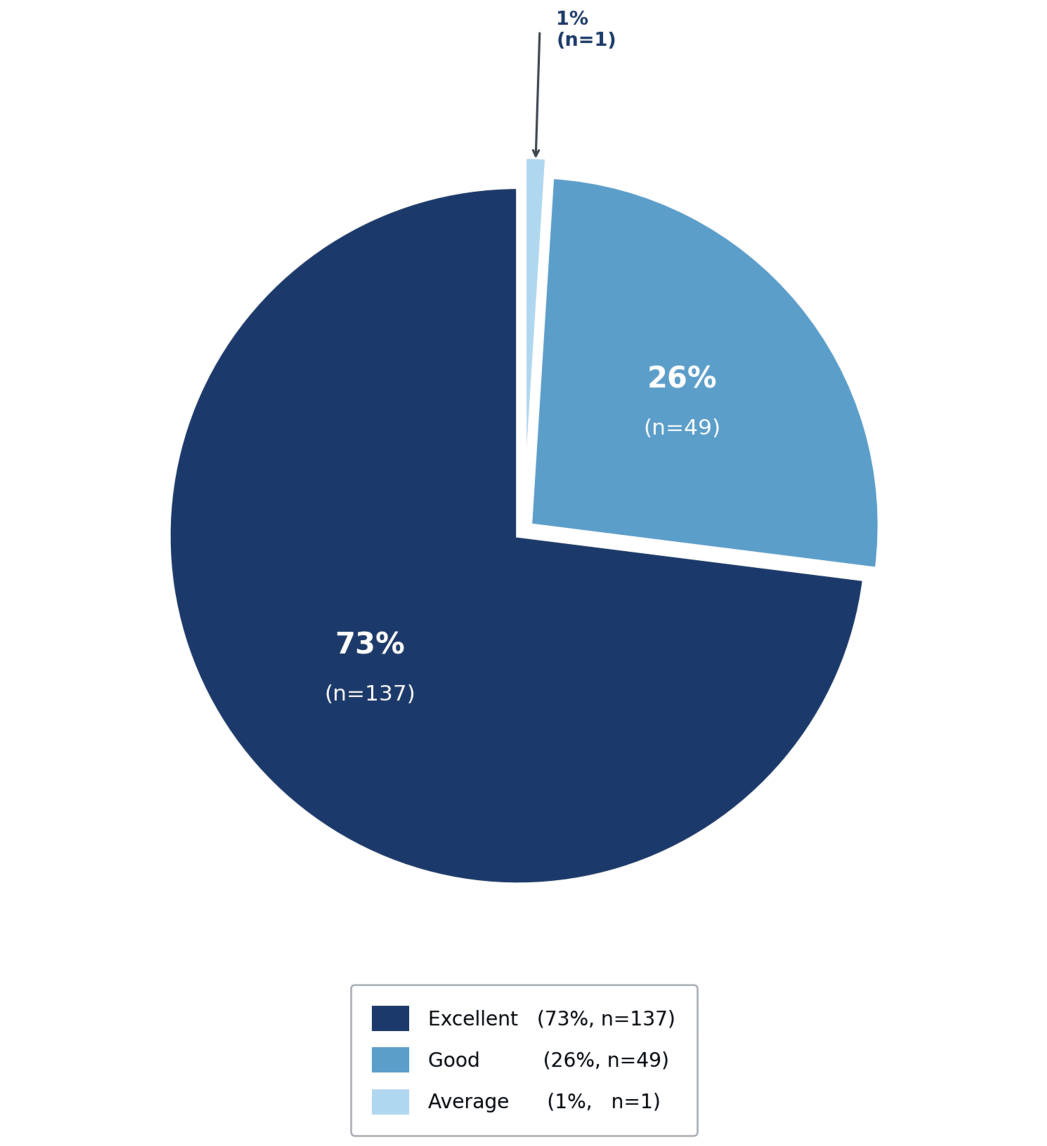
Overall ACLS training quality ratings among postgraduate residents (N = 187). The majority rated training as excellent (73%), followed by good (26%) and average (1%). ACLS: Advanced Cardiovascular Life Support

Descriptive statistics for the four primary outcome variables (all self-reported) are presented in Table 3. The mean confidence score was 9.75 ± 1.33 (95% CI: 9.55-9.94; range: 3-12), indicating a moderate-to-high level of self-reported confidence in applying ACLS protocols. The mean perceived clinical competence score was 22.83 ± 3.29 (95% CI: 22.35-23.31; range: 13-28). The mean monitoring and adherence score was 6.55 ± 1.59 (95% CI: 6.32-6.78), reflecting moderate adherence to recommended monitoring practices. The barriers score demonstrated the greatest variability, with a mean of 6.20 ± 3.63 (95% CI: 5.67-6.73; range: 0-14), indicating heterogeneous patterns of challenge across participants. Overall ACLS training quality was rated as excellent by 73% (n = 137) of participants, good by 26% (n = 49), and average by 1% (n = 1), as illustrated in Figure 1.

**Table 3:** Descriptive statistics of key study variables among postgraduate residents following ACLS training (N = 187). All values are self-reported. All scores are derived from self-reported questionnaire responses. SD: standard deviation; CI: confidence interval; ACLS: Advanced Cardiovascular Life Support

Variable	Mean ± SD	95% CI	Min	Max
Confidence score	9.75 ± 1.33	9.55-9.94	3	12
Monitoring & adherence score	6.55 ± 1.59	6.32-6.78	0	8
Barriers score	6.20 ± 3.63	5.67-6.73	0	14
Perceived clinical competence score	22.83 ± 3.29	22.35-23.31	13	28

Challenges encountered during resuscitation

**Figure 2:**
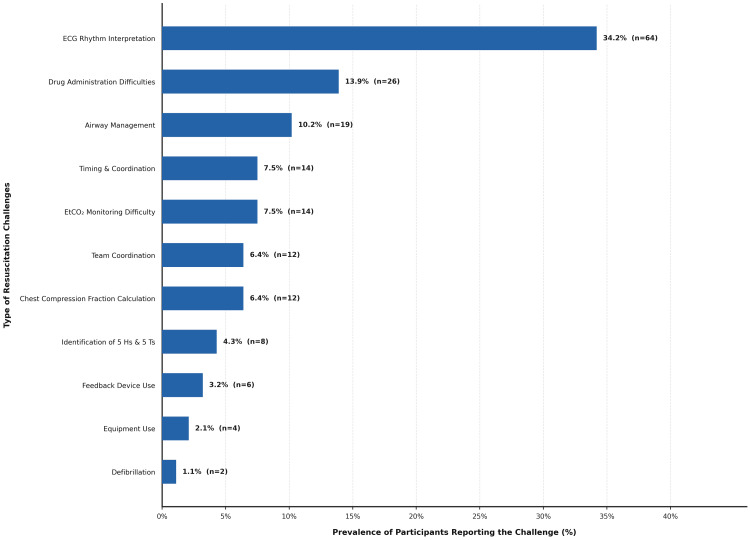
Resuscitation challenges among ACLS-certified postgraduate residents. Bar chart illustrating the frequency distribution of resuscitation challenges reported by postgraduate residents (N = 187). ECG rhythm interpretation was the most prevalent challenge (34.2%; n = 64), followed by drug administration difficulties (13.9%; n = 26) and airway management (10.2%; n = 19). EtCO₂: end-tidal carbon dioxide (7.5%; n = 14). ACLS: Advanced Cardiovascular Life Support; ECG: electrocardiogram; 5 Hs: hypovolemia, hypoxia, hydrogen ion (acidosis), hyperkalemia, and hypothermia; 5 Ts: tension pneumothorax, tamponade (cardiac), toxins, thrombosis (pulmonary), and thrombosis (coronary)

Participants were asked to identify challenges encountered during live resuscitation events across 11 predefined domains. ECG rhythm interpretation was the most prevalent challenge, reported by 64 participants (34.2%; 95% CI: 27.3-41.9%), representing more than twice the frequency of the second most common difficulty. Drug administration errors or difficulties were reported by 26 participants (13.9%), and airway management challenges were reported by 19 participants (10.2%). Timing and coordination difficulties, as well as EtCO₂ monitoring challenges, were each reported by 14 participants (7.5%). Team coordination and calculation of chest compression fraction were each reported by 12 participants (6.4%). Less frequently cited challenges included identification of reversible causes (5 Hs (hypovolemia, hypoxia, hydrogen ion (acidosis), hyperkalemia, and hypothermia) and 5 Ts (tension pneumothorax, tamponade (cardiac), toxins, thrombosis (pulmonary), and thrombosis (coronary)); n = 8; 4.3%), feedback device use (n = 6; 3.2%), equipment-related issues (n = 4; 2.1%), and difficulties with defibrillation (n = 2; 1.1%). The complete frequency distribution is summarized in Table 4 and illustrated in Figure 2.

**Table 4:** Frequency distribution of resuscitation challenges reported by postgraduate residents during in-hospital cardiac arrest resuscitation (N = 187), ranked by descending frequency. Participants were permitted to select all applicable challenges from 11 predefined domains (item Q17; multiple selections allowed). 95% CI values are presented for the proportion of participants reporting each challenge. EtCO₂: end-tidal carbon dioxide; CI: confidence interval; ECG: electrocardiogram

Resuscitation domain	n	%	95% CI lower	95% CI upper
ECG Rhythm Interpretation	64	34.2	27.3	41.9
Drug Administration Difficulties	26	13.9	9.3	19.8
Airway Management	19	10.2	6.3	15.5
EtCO₂ Monitoring Difficulty	14	7.5	4.2	12.3
Timing & Coordination	14	7.5	4.2	12.3
Team Coordination	12	6.4	3.4	11.0
Chest Compression Fraction Calculation	12	6.4	3.4	11.0
Identification of 5 Hs & 5 Ts	8	4.3	1.9	8.2
Feedback Device Use	6	3.2	1.2	6.9
Equipment Use	4	2.1	0.6	5.3
Defibrillation	2	1.1	0.1	3.8

Utilization of monitoring tools

**Figure 3:**
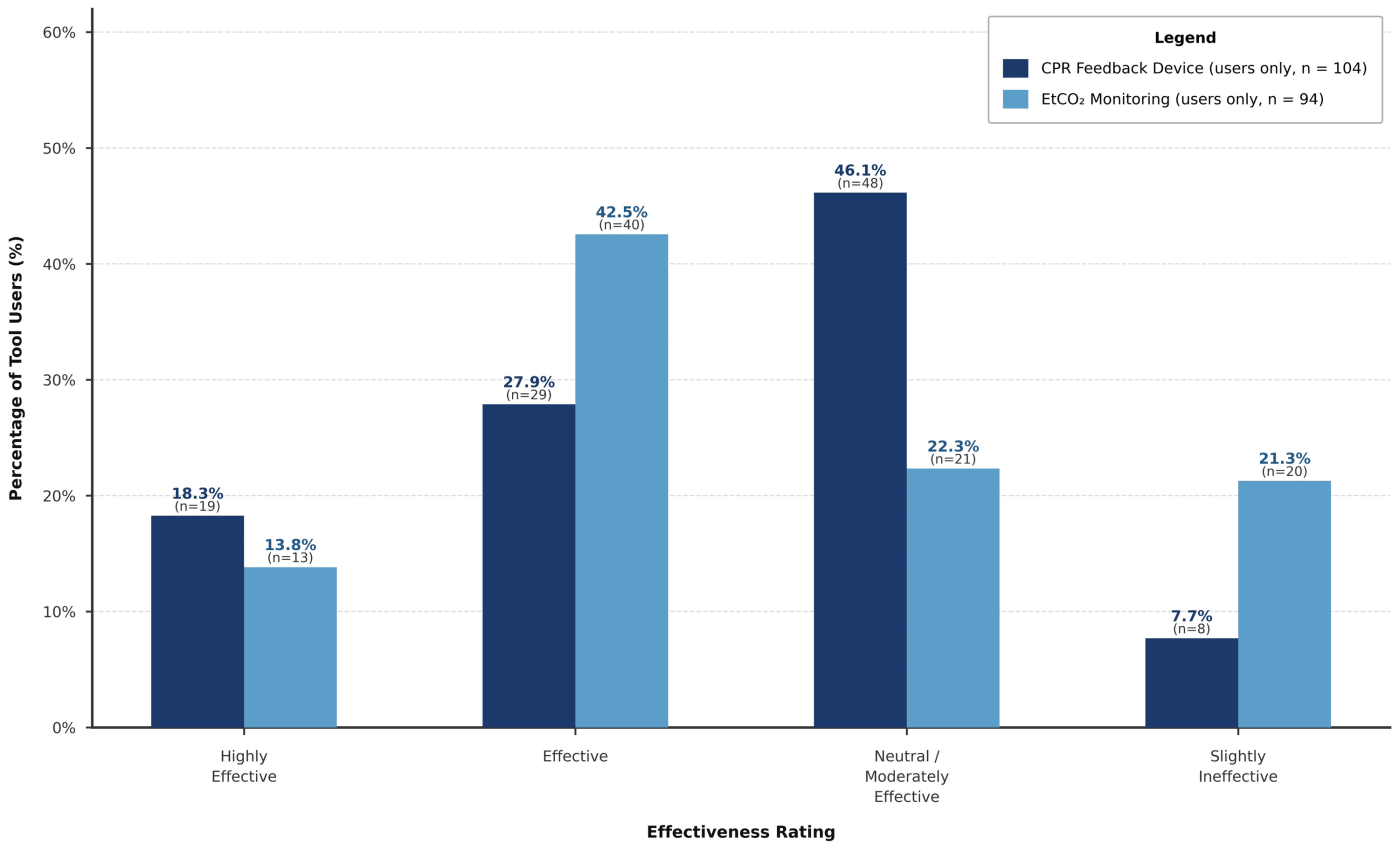
Effectiveness ratings of real-time CPR feedback devices and EtCO₂ monitoring among participants who used these tools. EtCO₂: end-tidal carbon dioxide; CPR: cardiopulmonary resuscitation

The AHA-recommended physiological monitoring tools assessed in this study were real-time audiovisual CPR feedback devices (Corpuls 3 Feedback monitor), which provide continuous feedback on compression rate, depth, and recoil, and EtCO₂ capnography monitors. The utilization of the two AHA-recommended physiological monitoring tools, real-time CPR feedback devices and EtCO₂ monitoring systems, during resuscitation events revealed notable gaps among participants. Of the 184 respondents who answered this item, 80 participants (43.5%) did not use real-time CPR feedback devices during resuscitations; among those who did use them, 46.1% (n = 48) rated their effectiveness as neutral. Similarly, of 183 respondents, 89 participants (48.9%) reported not utilizing EtCO₂ monitoring; of those who did use it, 22.3% (n = 21) found it moderately effective. These findings indicate that nearly half of the participants were not consistently applying AHA-recommended monitoring practices despite completing ACLS training. Figure 3 depicts a grouped bar chart showing effectiveness ratings for real-time CPR feedback devices and EtCO₂ monitoring among participants who used these tools. Among feedback device users, 46.1% (n = 48) rated effectiveness as neutral; among EtCO₂ users, 22.3% (n = 21) found it moderately effective. Table 5 outlines the utilization of AHA-recommended physiological monitoring tools such as real-time CPR feedback devices and EtCO₂ monitoring systems among PG residents.

**Table 5:** Utilization of AHA-recommended physiological monitoring tools during resuscitation events among postgraduate residents. Bold entries indicate used proportions. Missing responses represent participants who did not answer the relevant item. Percentages are valid percentages calculated only from respondents. AHA: American Heart Association; EtCO₂: end-tidal carbon dioxide; CPR: cardiopulmonary resuscitation

Monitoring tool	Used, n (%)	Not used, n (%)	Missing responses
Real-time CPR feedback device	104 (56.5%)	80 (43.5%)	3
EtCO₂ monitoring	94 (51.1%)	89 (48.9%)	4

It is important to acknowledge, however, that non-utilization of these monitoring tools may not be solely attributable to gaps in ACLS training. Practical barriers including equipment unavailability at the bedside, time-critical resuscitation dynamics that prioritize compressions and airway management over device set-up, institutional procurement limitations, and ward-level versus ICU-level resource disparities may independently contribute to monitoring tool non-use. The present study did not systematically assess equipment availability, institutional protocols governing monitoring tool use, or contextual factors at the time of resuscitation. Causal attribution of non-use solely to training gaps is therefore not warranted based on the available data. Future prospective studies should assess the relative independent contributions of training adequacy, device availability, and institutional policy to monitoring tool utilization rates during resuscitation.

The clinical magnitude of these findings warrants explicit emphasis. A non-utilization rate of 43.5% for real-time CPR feedback devices means that approximately one in every two resuscitation events in this cohort was conducted without objective, real-time verification of compression quality, directly risking deviation from AHA-recommended compression rates of 100-120 per minute and target depths of at least 5 cm. Similarly, the absence of EtCO₂ monitoring in 48.9% of resuscitations eliminates the earliest non-invasive physiological indicator of ROSC, potentially delaying recognition of ROSC and the timely initiation of post-resuscitation care. These are not merely statistical observations; they represent clinically significant gaps with direct implications for patient survival and neurological recovery outcomes following IHCA.

Assessment of normality

Prior to between-group comparisons, the Shapiro-Wilk test was applied to assess the distributional properties of the three primary outcome variables stratified by group. Results indicated significant deviation from normality for all variables in both the clinician and non-clinician subgroups (all p < 0.05), justifying the use of non-parametric statistical methods for group comparisons. Results are detailed in Table 6.

**Table 6:** Shapiro-Wilk normality test results stratified by group for key study variables. Significance of p < 0.05 confirms non-normal distribution, justifying the use of the Mann-Whitney U test for intergroup comparisons.

Variable	Group	W	df	p-value
Confidence score	Clinicians	0.902	132	<0.001
	Non-clinicians	0.838	53	<0.001
Perceived clinical competence score	Clinicians	0.951	132	<0.001
	Non-clinicians	0.937	53	0.008
Monitoring & adherence score	Clinicians	0.801	132	<0.001
	Non-clinicians	0.834	53	<0.001

Comparison between clinicians and non-clinicians

Mann-Whitney U tests revealed no statistically significant differences between clinicians and non-clinicians on any of the three primary outcome measures (Table 7). Median confidence scores were comparable between groups (Mdn = 10; interquartile range (IQR): 9-11 vs. 9-10; Z = −0.370, p = 0.712). Similarly, perceived clinical competence scores (Z = −0.452, p = 0.651) and monitoring and adherence scores (Z = −0.611, p = 0.541) did not differ significantly between groups. These findings indicate that post-ACLS training outcomes were equitable across clinical and non-clinical specialties at this institution.

**Table 7:** Mann-Whitney U test comparing confidence, perceived competence, and monitoring adherence between clinician and non-clinician postgraduate residents. A p-value > 0.05 indicates no statistically significant difference between groups. IQR: interquartile range

Variable	Clinicians median (IQR)	Non-clinicians median (IQR)	Z	p-value
Confidence score	10 (9-11)	10 (9-10)	−0.370	0.712
Perceived clinical competence score	22.5 (21-26)	22 (21-25)	−0.452	0.651
Monitoring & adherence score	7 (6-8)	7 (6-8)	−0.611	0.541

Correlation between confidence and perceived clinical competence

Spearman’s rank correlation analysis demonstrated a statistically significant, strong positive association between confidence score and perceived clinical competence score (ρ = 0.692, p < 0.001; N = 186), indicating that participants who reported higher self-confidence in performing ACLS procedures also reported markedly greater perceived competence in managing cardiac arrest scenarios (Table 8). This relationship held across both clinical and non-clinical subgroups.

**Table 8:** Spearman’s rank correlation between confidence score and perceived clinical competence score (N = 186). p < 0.001 indicates a statistically significant strong positive correlation. ρ: Spearman’s rho correlation coefficient; CI: confidence interval

Variable pair	Spearman ρ	95% CI	p-value
Confidence score ↔ perceived clinical competence score	0.692	0.609-0.760	<0.001

Predictors of perceived clinical competence: multiple linear regression

Multiple linear regression was performed with perceived clinical competence as the dependent variable and confidence score, barriers score, group (clinician/non-clinician), EtCO₂ monitoring use, and ECG interpretation difficulty as predictors. The overall model was statistically significant (F(5, 174) = 38.784, p < 0.001) and explained 52.7% of the variance in competence scores (R² = 0.527; adjusted R² = 0.513), indicating excellent explanatory power for a behavioral research model (Table 9).

**Table 9:** Multiple linear regression model predicting perceived clinical competence score among postgraduate residents. Dependent variable: perceived clinical competence score. B: unstandardized coefficient; SE: standard error; β: standardized coefficient. R² = 0.527; adjusted R² = 0.513; F(5, 174) = 38.784, p < 0.001. The Confidence score row represents the sole statistically significant predictor (p < 0.05); all other predictors were non-significant. ECG: electrocardiogram; EtCO₂: end-tidal carbon dioxide

Predictor	B	SE	β	t	p	95% CI
Constant	6.441	1.754	-	3.673	<0.001	2.98-9.90
Confidence score	1.791	0.134	0.724	13.417	<0.001	1.53-2.05
Barriers score	−0.107	0.057	−0.118	−1.888	0.061	−0.22-0.005
Group (clinician/non-clinician)	0.032	0.383	0.004	0.082	0.934	−0.72-0.79
Use of EtCO₂ monitoring	−0.515	0.891	−0.030	−0.578	0.564	−2.27-1.24
ECG interpretation difficulty	0.157	0.439	0.023	0.357	0.722	−0.71-1.02

Confidence score emerged as the sole statistically significant independent predictor of perceived clinical competence (B = 1.791, standard error (SE) = 0.134, β = 0.724, t = 13.417, p < 0.001; 95% CI: 1.53-2.05), indicating that each one-unit increase in confidence score was associated with a 1.791-unit increase in perceived competence, independent of other covariates. The barriers score showed a trend toward a negative association with competence (B = −0.107, β = −0.118, p = 0.061) that approached but did not reach statistical significance. Group classification, EtCO₂ monitoring use, and ECG interpretation difficulty were not significant predictors in the adjusted model (all p > 0.05).

## Discussion

This cross-sectional study systematically evaluated the impact of AHA-accredited ACLS training on the confidence, perceived clinical competence, guideline adherence, and resuscitation challenges of PG medical residents at a tertiary care teaching hospital. The principal findings indicate that participants reported high levels of confidence and perceived foundational resuscitation competence following ACLS training across both clinical and non-clinical specialties. However, significant and clinically meaningful deficiencies persist in ECG rhythm interpretation and physiological monitoring tool utilization, and the integration of ACLS competencies into complex live resuscitation scenarios remains challenging for a notable proportion of residents.

Confidence and perceived clinical competence

Participants demonstrated moderate-to-high post-training confidence scores (mean: 9.75 ± 1.33), and a strong positive correlation was observed between confidence and perceived clinical competence (ρ = 0.692, p < 0.001). These findings are consistent with published evidence suggesting that structured simulation-based training is associated with higher self-efficacy and perceived preparedness for emergency scenarios following training [5,6]. Importantly, confidence score was the only significant independent predictor of perceived competence in the regression model (β = 0.724, p < 0.001; R² = 0.527), suggesting that cultivating trainee confidence through repeated exposure to realistic resuscitation scenarios may be as important a training objective as the transmission of cognitive knowledge. This has implications for curriculum design: simulation-based platforms should not only assess competency but should be structured to support and sustain trainee confidence through graduated challenge and constructive feedback.

It is important to note, however, that self-reported confidence does not necessarily equate to objective clinical competence. The Dunning-Kruger effect, wherein lower-performing individuals tend to overestimate their abilities, has been documented among medical residents, including in Emergency Medicine contexts [17]. The reliance on self-reported outcomes in this study means that the confidence-competence association observed here should be interpreted with caution. Future studies using objective performance metrics during simulated or actual resuscitations are necessary to validate these self-reported findings.

ECG interpretation: the most prevalent resuscitation challenge

ECG rhythm interpretation was identified as the most frequently reported challenge during live resuscitations, affecting 34.2% of participants, more than twice the prevalence of any other difficulty. This finding aligns with well-established literature documenting persistent ECG interpretation deficiencies among medical residents and ACLS-certified providers [9]. Kashou et al. demonstrated that proficiency in ECG interpretation is not universal among healthcare providers, noting that a significant proportion of residents and practicing clinicians fail to meet established competency thresholds, with barriers rooted at multiple levels of medical curricula [9]. The prevalence of ECG interpretation difficulty identified in the present study (34.2%) is notably higher than rates reported in comparable PG training contexts, further emphasizing the urgent requirement for a dedicated, competency-assessed ECG module within the ACLS curriculum [9]. The findings of the present study provide further support for the integration of dedicated ECG interpretation training, ideally a structured, competency-assessed module of at least six hours, into the ACLS pre-course or residency curriculum.

Utilization of physiological monitoring tools

In line with previously documented patterns of underutilization, this study identified significant gaps in the adoption of physiological monitoring tools during resuscitation events. Nearly half of the participants did not use real-time CPR feedback devices (43.5%) or EtCO₂ monitoring (48.9%). Among participants who used CPR feedback devices, 46.1% rated their effectiveness as neutral, and among those who used EtCO₂ monitoring, 22.3% found it only moderately effective. These observations suggest that familiarity with, and clinical confidence in, interpreting these tools in real-time scenarios remains an area requiring targeted reinforcement. However, it is important to acknowledge that other factors beyond training-related gaps in knowledge or clinical confidence may contribute to the non-utilization of EtCO₂ monitoring and real-time CPR feedback devices during resuscitation events. Institutional and system-level factors, including equipment availability at the point of care, device maintenance and procurement constraints, the logistical demands of emergency resuscitation, and departmental policies governing monitoring tool deployment, may independently contribute to underutilization. As this study did not systematically assess the reasons for non-use, causal attribution to training deficits alone would be an overinterpretation of the available data. Future studies should include structured assessment of equipment availability and institutional policy adherence alongside self-reported utilization patterns to enable more precise identification of the determinants of monitoring tool adoption in clinical resuscitation practice.

This is clinically significant because EtCO₂ monitoring has been shown to be a strong indicator of coronary and cerebral perfusion pressure during CPR and is the first non-invasive sign of ROSC [10,11]. The landmark study by Abella et al. demonstrated that real-time audiovisual CPR feedback systems significantly improved the consistency of chest compression rates and ventilation delivery during IHCA, with guideline-compliant compression rates increasing from 22% to 72% following feedback device implementation [12]. Furthermore, adherence to the recommended chest compression rate is closely linked to survival outcomes. Idris et al. found that compression rates outside the AHA-recommended range of 100-120 compressions per minute were associated with lower rates of ROSC, further underscoring the importance of real-time CPR feedback devices in maintaining guideline-compliant resuscitation [18]. In the present study, 43.5% of participants did not use CPR feedback devices during resuscitation, and among those who did, 46.1% rated their effectiveness as only neutral findings, which suggests that even when these devices are available, their perceived clinical benefit remains suboptimal, possibly reflecting insufficient hands-on training with these tools during the ACLS curriculum.

Intergroup comparisons

There were no statistically significant differences in confidence, perceived competence, or adherence to monitoring between clinicians and non-clinicians (all p > 0.05). This finding challenges the intuitive assumption that clinical specialty directly determines resuscitation readiness and suggests that structured, standardized ACLS training may effectively equalize foundational competency across diverse professional backgrounds. This has potential implications for the universal mandatory inclusion of ACLS training across all PG medical specialties, regardless of direct clinical exposure to cardiac arrest.

Broader resuscitation challenges

Beyond ECG interpretation, participants reported a range of challenges reflecting the multifaceted complexity of IHCA management. Drug administration difficulties (13.9%) and airway management challenges (10.2%) represent areas where procedural competency under time pressure and cognitive load may benefit from targeted simulation reinforcement. Team coordination difficulties (6.4%) and timing and coordination challenges (7.5%) highlight the importance of interdisciplinary, team-based resuscitation training that specifically addresses closed-loop communication, role delineation, and task synchronization under simulated high-pressure conditions. Environmental stressors, such as ward noise, spatial limitations, and equipment inaccessibility, are intrinsic characteristics of actual IHCA incidents and can only be effectively anticipated through high-fidelity simulation.

Recommendations for ACLS curriculum enhancement

Based on the findings of this study, the following evidence-based recommendations are proposed (summarized in Table 10).

**Table 10:** Evidence-based recommendations for ACLS curriculum enhancement based on study findings. ECG: electrocardiogram; ACLS: Advanced Cardiovascular Life Support; EtCO₂: end-tidal carbon dioxide; CPR: cardiopulmonary resuscitation

Recommendation	Rationale and description
1. Structured biannual refresher training	Low-dose, high-frequency refresher programs emphasizing ECG rhythm recognition, physiological monitoring, and CPR quality should be mandated between formal recertification cycles. These have demonstrated superiority over annual or biennial formats in sustaining cognitive and psychomotor skill levels [6].
2. Dedicated ECG interpretation module	A structured, competency-assessed ECG course integrated into the ACLS curriculum, with a minimum duration of six hours, should address the persistent skill gap in rhythm recognition identified in this and prior studies [8].
3. High-fidelity simulation-based learning	Regular, mandatory cardiac arrest simulation incorporating EtCO₂ monitoring and real-time CPR feedback devices should be embedded in postgraduate training programs to develop device familiarity and procedural confidence before clinical deployment [9-11].
4. Interdisciplinary resuscitation drills	Multidisciplinary team training exercises involving medical, nursing, and allied health staff should be conducted regularly to reinforce communication, delineate team roles, and rehearse coordinated execution of resuscitation protocols.
5. Mandatory integration of monitoring technology	Institutional policies should mandate the use of EtCO₂ monitoring and real-time CPR feedback devices in all in-hospital cardiac arrest events, supported by systematic training, equipment procurement, and quality-improvement oversight [9-11].
6. Post-resuscitation debriefing	Structured, facilitated post-resuscitation debriefing sessions should be conducted routinely to reinforce learning, identify system-level gaps, and promote continuous quality improvement in resuscitation practice.

Limitations

This study has several limitations that merit consideration. First, this study relies exclusively on self-reported measures of confidence and perceived competence, which introduces several important sources of bias. Social desirability bias may have led participants to overreport guideline adherence and underreport clinical challenges, resulting in an overestimation of actual competence levels. Recall bias is a further concern, as participants were asked to retrospectively report experiences from resuscitation events occurring over the preceding six months, during which accurate recollection of specific clinical details may have been incomplete or distorted. Additionally, medical education research has well recognized the potential for overestimating competence. The Dunning-Kruger effect, in which lower-performing individuals systematically overestimate their abilities, has been specifically documented among Emergency Medicine residents [17]. Taken together, these biases suggest that the confidence and competence scores reported in this study should be interpreted as reflections of subjective, perceived preparedness rather than objective clinical proficiency. Objective performance measures such as direct observation or video-based review of resuscitation events would provide a more externally valid assessment of provider competency [7].

Second, the single-center design represents a significant limitation with respect to external validity. As this study was conducted exclusively at an AHA-accredited tertiary care institution with dedicated simulation infrastructure, structured ACLS curricula, and mandatory PG training requirements, the levels of confidence and perceived competence observed may not be representative of PG residents training at non-accredited institutions, government medical colleges, lower-resource settings, or institutions with variable training quality and equipment availability. Specifically, the high proportion of participants rating ACLS training quality as excellent (73%) may reflect the superior training infrastructure at this institution rather than a generalizable finding across Indian medical institutions. Similarly, monitoring tool non-utilization rates may differ substantially in settings where EtCO₂ monitors and CPR feedback devices are not routinely available. To enhance generalizability, future studies should adopt multicenter designs encompassing institutions with varying accreditation status, resource levels, urban versus rural settings, and geographic diversity across India and comparable low- and middle-income country contexts. Additionally, the use of standardized objective performance assessments rather than self-report instruments would further strengthen cross-institutional comparability of findings.

Third, the absence of a pre-training baseline assessment or a control group precludes direct attribution of observed competency levels to the ACLS intervention. The cross-sectional design precludes the evaluation of skill retention trajectories over time; consequently, the findings should be regarded as indicative of post-training perceived competence at a singular time point, rather than as evidence of training-induced enhancement.

Fourth, a further limitation concerns the selection bias introduced by the inclusion criterion requiring participation in at least one IHCA resuscitation event within six months of ACLS certification. Residents who had not encountered a resuscitation event during this period, potentially those rotating through less acute or non-clinical specialties, were excluded from the analysis. This may have resulted in a study sample with systematically higher clinical exposure and consequently higher confidence and perceived competence scores than would be observed in the broader PG resident population. The findings should therefore be interpreted as reflecting residents with at least some real-world resuscitation exposure and may not generalize to recently certified ACLS providers who have not yet participated in a cardiac arrest event. Future multicenter studies incorporating longitudinal follow-up, objective performance metrics, and standardized outcome measures are warranted [9].

## Conclusions

Among PG medical residents who completed AHA-accredited ACLS training, self-reported confidence and perceived clinical competence were notably high, suggesting that the program lays a solid perceived foundation for emergency cardiovascular care. That said, given the cross-sectional nature of this study and the absence of pre-training baseline data, these findings reflect post-training perceptions rather than objectively measured improvements attributable to the intervention. Nearly half of the participants did not use EtCO₂ monitoring or real-time CPR feedback devices during actual resuscitations, pointing to a clear need for more deliberate integration of these tools into both training and clinical practice. ECG rhythm interpretation remained the single most common challenge encountered, and confidence was the strongest predictor of perceived competence, findings that together call for structured curricular reforms, including dedicated ECG modules, biannual simulation-based refresher training, and mandatory adoption of physiological monitoring technology. Addressing these gaps in a systematic and sustained manner holds meaningful promise for strengthening resuscitation preparedness among PG residents. Future research should pursue multicenter, longitudinal designs with objective performance measures to build on these preliminary findings.

## Figures and Tables

**Figure 4:**
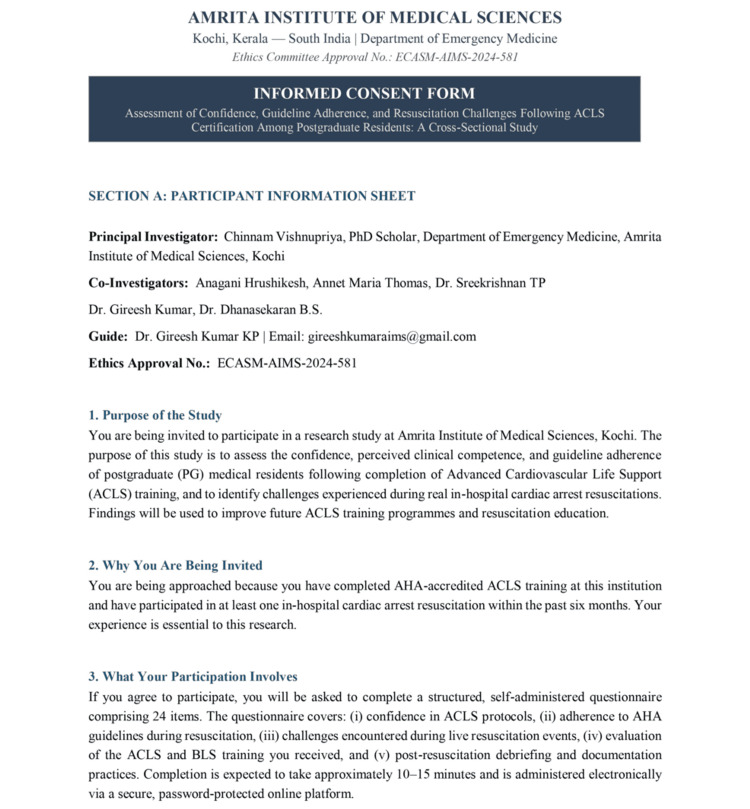
Informed consent form. Page No. 01

**Figure 5:**
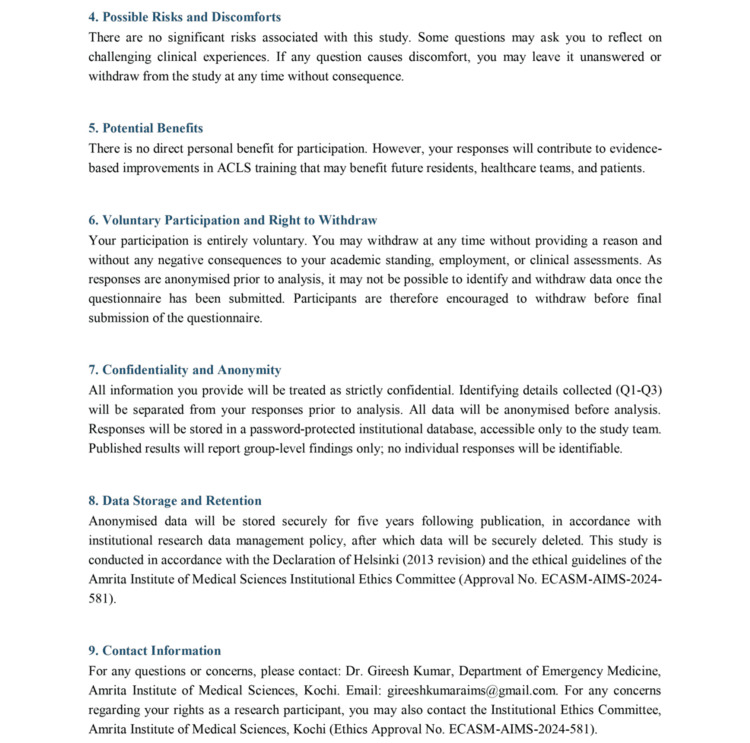
Informed consent form. Page No. 02

**Figure 6:**
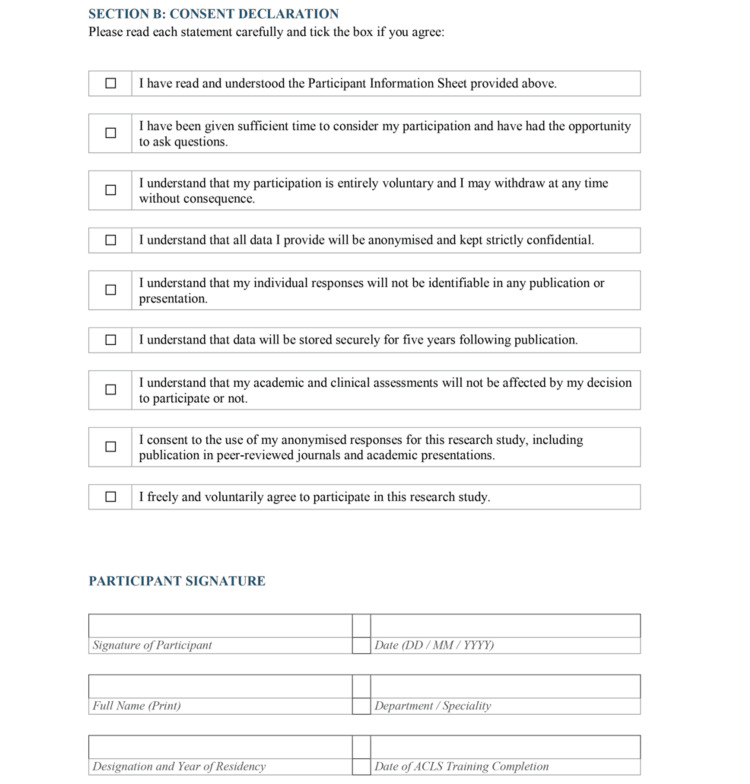
Informed consent form. Page No. 03

**Figure 7:**
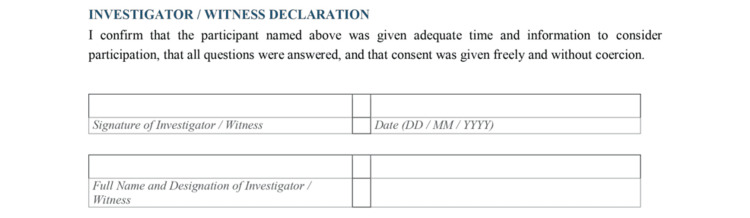
Informed consent form. Page No. 04

**Figure 8:**
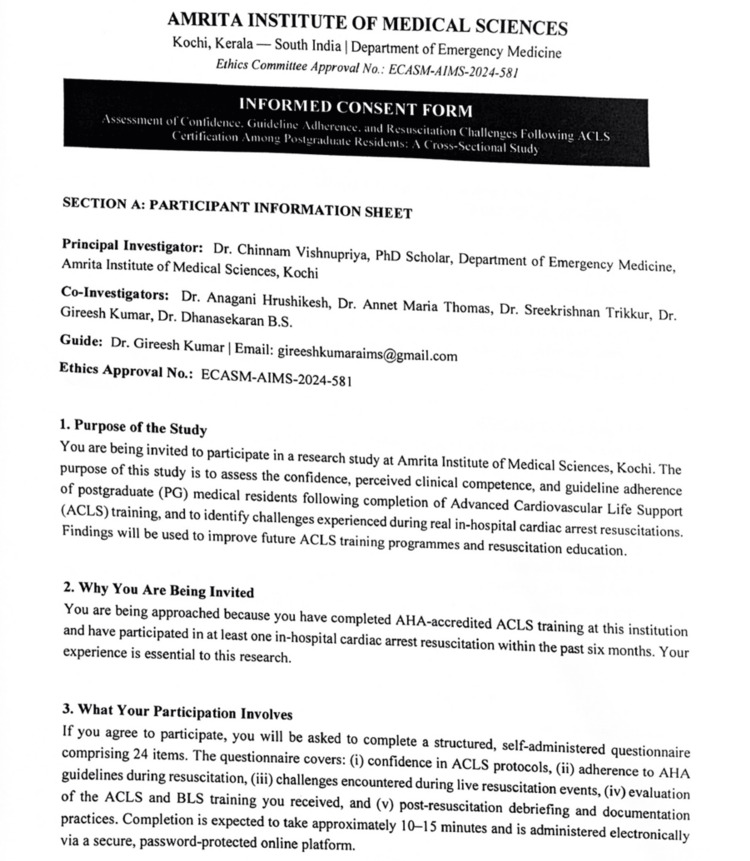
Consent form-signed copy. Page 01

**Figure 9:**
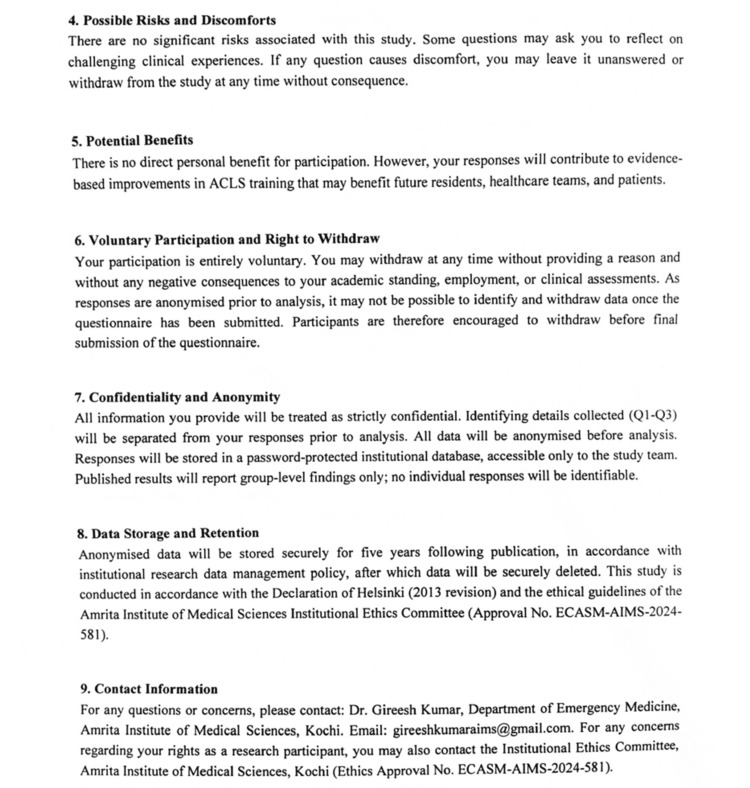
Consent form-signed copy. Page 02

**Figure 10:**
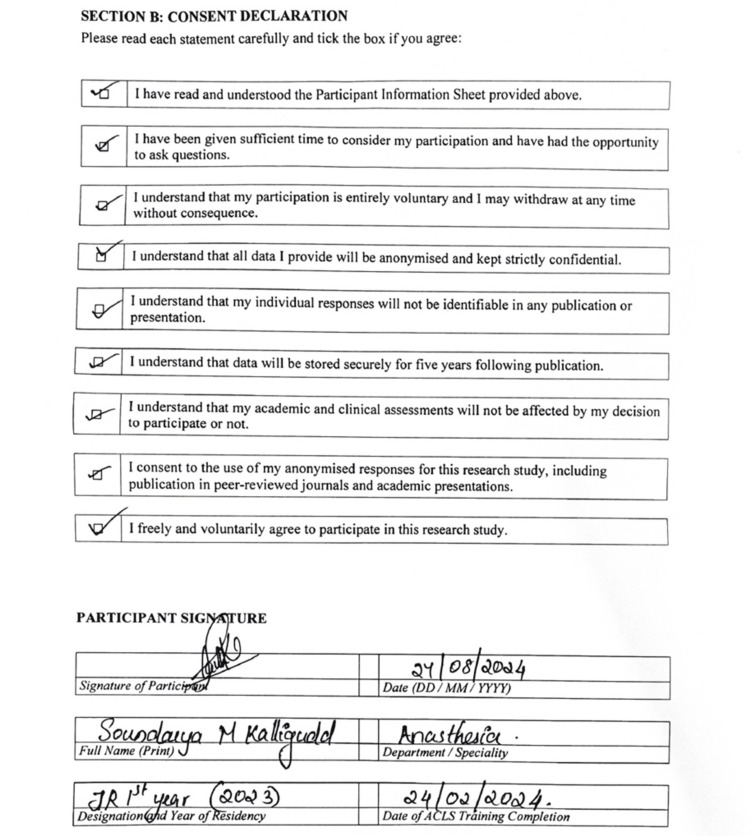
Consent form-signed copy. Page 03

**Figure 11:**
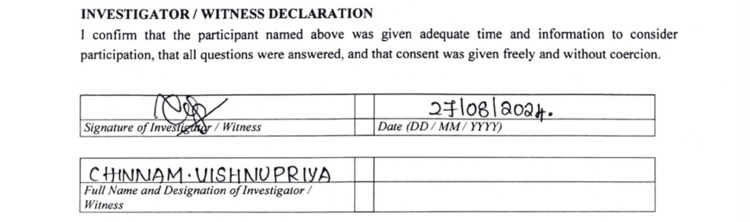
Consent form-signed copy. Page 04

## Appendices

Appendix A

Participant Information-

All fields are mandatory. Responses are anonymized prior to analysis.

Q1. Name

Q2. Designation and year

Q3. Department

Q4. Date of ACLS training completion (DD/MM/YYYY)

Domain 1: Confidence in ACLS Application (Questions 5-9)

For each question, mark only one oval to indicate your level of confidence or proficiency.

Q5. How confident do you feel in applying ACLS protocols during a real-life emergency situation?

○ Very confident

○ Confident

○ Neutral

○ Not confident

○ Not at all confident

Q6. To what extent did the ACLS training prepare you for actual resuscitation scenarios?

○ Extremely well

○ Well

○ Moderately well

○ Poorly

○ Very poorly

Q7. How effectively can you perform airway management techniques?

○ Excellent: highly effective and efficient

○Good: generally effective with minor areas for improvement

○ Average: adequate with noticeable areas needing improvement

○Poor: significant challenges and limited effectiveness

○Very poor: major difficulties with airway management

Q8. How confident are you in interpreting ECGs during resuscitation?

○ Very confident

○ Confident

○ Neutral

○ Not at all confident

Q9. How proficient are you with the use of defibrillators, including the application of appropriate energy levels?

○ Excellent: fully confident in using defibrillators and adjusting energy levels

○ Good: generally effective but with occasional need for guidance

○ Average: basic competence with room for improvement

○ Poor: difficulties in using defibrillators effectively

○ Very poor: major challenges with defibrillation

Domain 2: Protocol Adherence (Questions 10-16)

Indicate your level of adherence to AHA resuscitation guidelines during actual cardiac arrest events.

Q10. How well can you administer and manage medications during resuscitation, including dosage calculations and timing?

○ Excellent: proficient in drug administration and management

○ Good: generally effective but occasionally needs support

○ Average: adequate with some areas needing improvement

○ Poor: difficulties with drug administration and management

○ Very poor: major challenges with medication administration

Q11. How effectively do you collaborate and communicate with your team during resuscitation efforts?

○ Excellent: seamless teamwork and communication

○ Good: generally effective teamwork with minor communication issues

○ Average: adequate teamwork with noticeable communication challenges

○ Poor: struggles with teamwork and communication

○ Very poor: major difficulties in working with the team

Q12. Did you use any feedback devices (e.g., real-time feedback for chest compressions)? If yes, how helpful were they?

○ Very helpful

○ Helpful

○ Neutral

○ Not helpful

○ Did not use

Q13. How effective was the use of EtCO₂ monitoring in assessing ventilation and resuscitation effectiveness?

○ Very effective

○ Effective

○ Neutral

○ Ineffective

○ Did not use

Q14. How confident are you in calculating and applying the chest compression fraction during resuscitation?

○ Very confident

○ Confident

○ Neutral

○ Not confident

○ Not at all confident

○ Not calculating at all

Q15. How well can you identify and treat the 5 Hs (hypovolemia, hypoxia, hydrogen ion (acidosis), hyperkalemia, and hypothermia) and 5 Ts (tension pneumothorax, tamponade (cardiac), toxins, thrombosis (pulmonary), and thrombosis (coronary)) during resuscitation?

○ Excellent: fully capable of identifying and treating all Hs and Ts

○ Good: generally capable but may need occasional support

○ Average: basic understanding with room for improvement

○ Poor: struggles with identification and treatment

○ Very poor: major difficulties in addressing Hs and Ts

Q16. How well were you able to manage timing and prioritize interventions during resuscitation?

○ Excellent: effective timing management and prioritization

○ Good: generally effective with minor timing issues

○ Average: adequate timing management with noticeable areas for improvement

○ Poor: struggles with timing management and prioritization

○ Very poor: major difficulties in managing timing and prioritization

Domain 3: Barriers and Challenges (Questions 17-18)

Select all options that apply for Item 17. Mark only one oval for Item 18.

Q17. Did you face any challenges while performing resuscitation procedures? (Select all that apply)

○ Airway management

○ ECG rhythm interpretation

○ Defibrillation

○ Drug administration

○ Team coordination

○ Feedback device use

○ EtCO₂ monitoring

○ Calculation of chest compression fraction (CCF)

○ Identification and treatment of 5 Hs and 5 Ts

○ Equipment use

○ Timing and coordination

Other (please specify): ___________________________

Q18. How did these difficulties affect the outcome of the resuscitation?

○ Significantly impacted: major delays or issues that adversely affected the outcome

○ Moderately impacted: some delays or issues that affected the outcome to a certain extent

○ Slightly impacted: minor delays or issues with minimal impact on the outcome

○ Did not impact: no noticeable effect on the resuscitation outcome

Domain 4: Training Effectiveness (Questions 19-23)

Questions 19, 20, 22, and 23 require a written response. Mark only one oval for Question 21.

Q19. What aspects of the ACLS training were most beneficial for you?

Q20. What areas of the training could be improved to enhance its effectiveness?

Q21. Did you find the BLS training to be sufficient and complementary to the ACLS training?

○ Yes

○ No

○ Somewhat

Q22. Do you have any recommendations for improving ACLS and BLS training programs?

Q23. Please provide any additional comments or suggestions for improving ACLS and BLS training.

Domain 5: Debriefing, Documentation, and Overall Rating (Items 24-28)

Mark only one oval per item.

Q24. How often do you debrief bystanders (family members, witnesses) after a cardiac arrest resuscitation?

○ Always

○ Often

○ Sometimes

○ Rarely or never

Q25. How consistently do you ensure thorough documentation of all critical details following a cardiac arrest event?

○ Always

○ Frequently

○ Occasionally

○ Rarely or never

Q26. How would you rate the overall quality of the ACLS training you received?

○ Excellent

○ Good

○ Average

○ Poor

○ Very poor

Q27. How likely are you to recommend this ACLS training program to a colleague or peer?

○ Very likely

○ Likely

○ Neutral

○ Unlikely

○ Very unlikely

Appendix B

Appendix C 
